# The PARTY program: a systematic approach to injury prevention for young road users around the world

**DOI:** 10.5249/jivr.v7i2.561

**Published:** 2015-07

**Authors:** Thomas Brockamp, Paola Koenen, Hendrik Wyen, Manuel Mutschler, Arasch Wafaisade, Marc Maegele, Thomas Paffrath, Christian Probst, Bertil Bouillon

**Affiliations:** ^*a*^Department of Traumatology and Orthopedic Surgery, Cologne-Merheim Medical Center (CMMC), University of Witten/ Herdecke, Cologne, Germany.; ^*b*^Department of Trauma, Hand and Reconstructive Surgery, Hospital of the Johann Wolfgang ,Goethe-University, Frank-furt, Germany.

Trauma remains the number one cause of death for the youngest half of the population and is responsible for more productive years of life lost than cancer, stroke, and heart disease combined.^1, 2^ In most regions of the world this epidemic of road traffic injuries is still increasing. In example, a total of 15.482 individuals died from road traffic accidents in Iran between 1999 and 2000.^3^ To decrease the number of accidents and death among all ages around the World the World Health Organization (WHO) focuses on this problem and set up the „Decade of Action for Road Safety 2011-2020“ in May 2011.

We like to introduce a 1-day in-hospital injury awareness and prevention program for youth aged 15 years and older called: The P.A.R.T.Y. program (Prevent Alcohol and Risk-Related Trauma in Youth).^4^ It was first set up in 1986 in Canada to act against the high and increasing number of road traffic victims. The program brings together groups within the hospital, from external agencies and victims of previous injuries, exposing youth to the potential physical and psychological impact that results from a traumatic injury. As the first center in the European Union (EU) this systematic approach to injury prevention was implemented into a trauma center in Germany in 2011. 

In a three month proof-of-concept we invited three different school classes to join the P.A.R.T.Y. program at our trauma center over a period of three month (one class per month) and raised a questionnaire including 6 different questions about the setting of our program and additionally we wanted all students to grade each station of the program. Fifty-eight students between fifteen and sixteen years of age attended the program during the pilot strategy in 2011. After attending the program the students evaluated it by giving each part of the trauma circle a grade between 1 and 6 (1 = best grade, 6 = worst grade). 58% (n= 34) of all students rated the program best grade (1) and 41 % (n= 24) with second grade. Grades between three and six were not awarded by the students who attended the program. 

Injury prevention strategies are shown to reduce the number of road traffic injuries in youth. 

The program is already part of injury prevention strategies in North America, Australia, Japan and Brazil. We proofed, that this program can also be set up in a level one trauma unit in Germany. We think that the P.A.R.T.Y. program can be integrated into the daily routine of trauma centers around the world to address the important topic of injury prevention.

## References

[1] Campbell BJ (1992). Reducing traffic injury: size of the problem and lack of research resources. World J Surg.

[2] Toroyan T (2009). Global status report on road safety. Inj prev.

[3] Montazeri A (2004). Road-traffic-related mortality in Iran: a descriptive study. Public Health.

[4] McDowall A. Prevent Alcohol and Risk Related Trauma in Youth - Report 2011. Report for the Sunnybrooks HSC Toronto.Toronto: College Health Sciences Centre, 2011:3-14.

